# A cancer-specific activatable theranostic nanodrug for enhanced therapeutic efficacy via amplification of oxidative stress

**DOI:** 10.7150/thno.39412

**Published:** 2020-01-01

**Authors:** Xie-an Yu, Mi Lu, Yingping Luo, Yiting Hu, Ying Zhang, Zhiming Xu, Shuaishuai Gong, Yunhao Wu, Xiao-Nan Ma, Bo-Yang Yu, Jiangwei Tian

**Affiliations:** State Key Laboratory of Natural Medicines, Jiangsu Key Laboratory of TCM Evaluation and Translational Research, Research Center for Traceability and Standardization of TCMs, Cellular and Molecular Biology Center, School of Traditional Chinese Pharmacy, China Pharmaceutical University, Nanjing 211198, P.R. China.

**Keywords:** cancer, amplification of oxidative stress, theranostics, dihydroartemisinin, nanodrug

## Abstract

**Rationale:** Despite considerable advances, the reactive oxygen species (ROS)-mediated cancer treatment suffers from the problems of up-regulation of adaptive antioxidants in cancer cells as well as side effects to normal cells. Therefore, development of a new generation of cancer-specific nanomedicine capable of amplifying oxidative stress would be of great interest for accurate and effective cancer treatment.

**Methods:** Herein, transferrin (Tf)-decorated, dihydroartemisinin (DHA), *L*-buthionine-sulfoximine (BSO), and CellROX-loaded liposomal nanoparticles (Tf-DBC NPs) were developed for precise cancer theranositcs. Tf-DBC NPs could specifically recognize cancer cells via Tf-Tf receptor binding and be uptaken into the lysosomes of cancer cells, where Tf-DBC NPs were activated to release Fe(II), DHA, and BSO. ROS was generated by DHA in the presence of Fe(II), and GSH was depleted by BSO to disrupt the redox balance in cancer cells. Furthermore, CellROX, as a fluorescent probe for imaging of intracellular oxidative stress, was used to monitor the therapeutic efficacy.

**Results:** The integration of Tf, DHA, and BSO into the acidic pH-responsive liposomes selectively and effectively killed cancer cells and prevented the oxidative injury to normal cells. The high oxidative state was visualized at the tumor site and the amplification of oxidative stress enabled tumor eradication by Tf-DBC NPs, demonstrating the successful implementation of this novel strategy *in vivo*.

**Conclusion:** Our study provides a new paradigm for the design of ROS-mediated therapeutics and offers a promising perspective for precise cancer treatment.

## Introduction

Oxidation therapy of cancers is an attractive strategy involved in chemotherapy, radiotherapy, and photodynamic therapy 1-7. These treatments kill cancer cells by *in situ* generation of reactive oxygen species (ROS) which are deleterious to cells due to their destructive actions on DNA, proteins, lipids, and other cellular constituents, resulting in the initiation and progression of dysfunctions and eventual cell death 8-14. However, cancer cells adapt to oxidative stress by activating adaptive antioxidants such as glutathione (GSH), to protect cells against endogenous and exogenous oxidative stress, which leads to survival and progression of cancer cells 12-22. Therefore, the design of a strategy that increases the oxidative stress and simultaneously depletes antioxidant species may efficiently kill cancer cells.

Recently, the amplification of oxidative stress strategy has been increasingly utilized in cancer treatment 23-30. Chen et al. prepared a MnO_2_-based nanoagent to enhance chemodynamic therapy by Fenton-like ion delivery to convert endogenous H_2_O_2_ into the highly toxic hydroxyl radical (•OH) and glutathione depletion 3. Ge and colleagues constructed a nanoreactor by incorporating Fe_3_O_4_ and glucose oxidase into a polyprodrug-based vesicule for cooperative cancer therapy 31. Liu and co-workers also prepared a nanocomplex by integrating Fenton catalyst and glutathione inhibitor to enhance cancer chemotherapy and radiotherapy 32. However, the inorganic or metallic nature of the nanomaterials like MnO_2_ and Fe_3_O_4_, as well as the lack of active targeting ability of these nanotherapeutics, raise concerns about their potential toxicity to normal tissues. These limitations have driven the future development of novel nanodrug with the properties of biocompatibility and tumor-specific activatable amplification of oxidative stress against cancer cells.

Transferrin (Tf) receptor is over-expressed on the surface of cancer cells providing an opportunity for cancer cell-specific recognition and targeted delivery by using Tf as a targeting ligand 33, 34. Also, due to the acidic environment of lysosomes in cancer cells, Fe(III) conjugated on Tf can be released and further reduced to Fe(II) by ferri reductase 35. Interestingly, Fe(II) has been demonstrated to be an effective catalyst to break the endoperoxide bridge of dihydroartemisinin (DHA) to generate abundant ROS increasing the intracellular oxidative levels 36, 37. In this process, Tf can play dual functions as a pilot for targeting Tf receptor overexpressed on tumor cells and as a ferric ion carrier for supplementing Fe(II) to catalyze DHA. Furthermore, *L*-buthionine-sulfoximine (BSO), as a potent inhibitor of GSH synthesis, can be used to further decrease the reductive levels of cancer cells 38. It is, therefore, rational to deduce that cancer cells would become extremely vulnerable if Tf, DHA, and BSO are integrated into one nanoparticle in an oxidative stress amplification strategy.

**Scheme 1:**
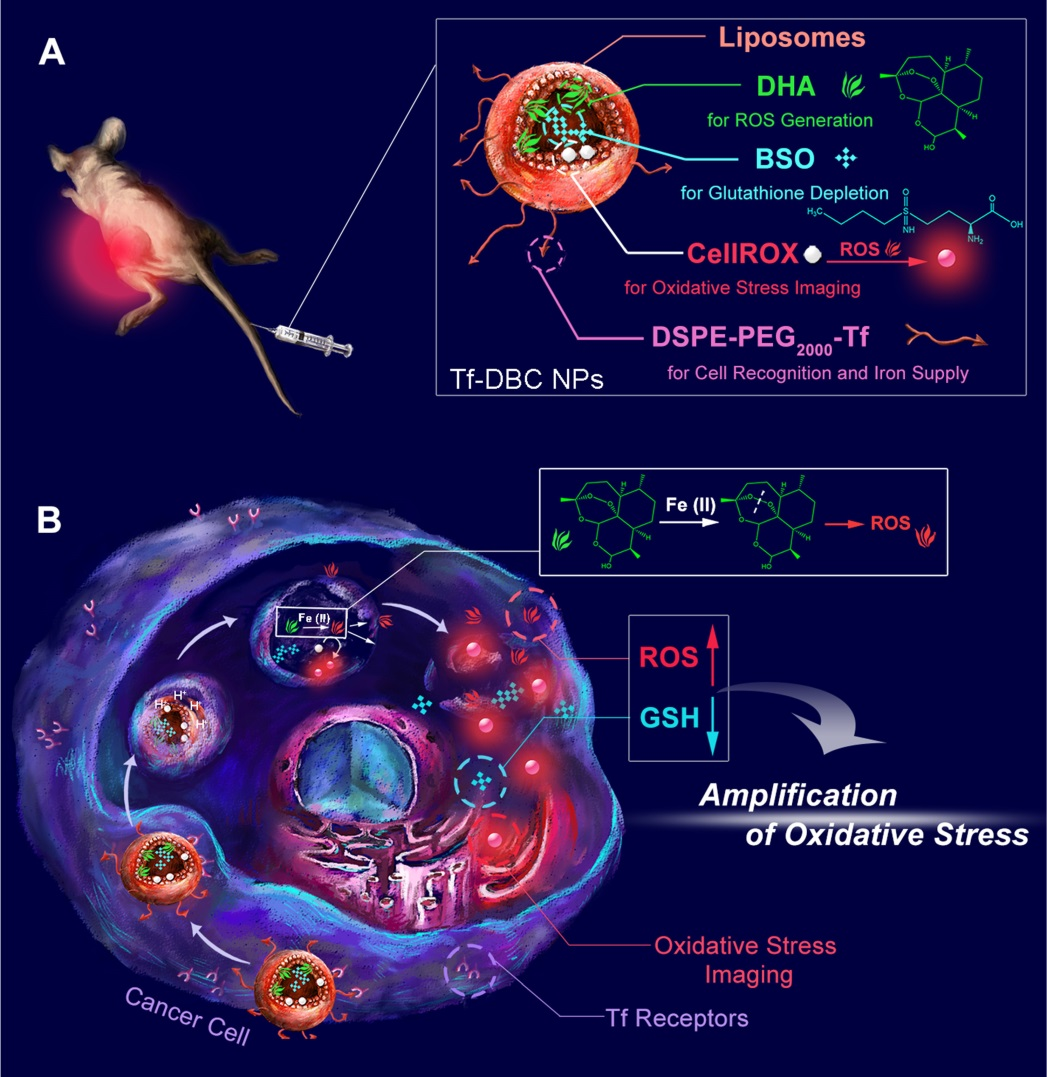
Schematic illustrations of (A) structure and (B) function of the Tf-DBC NPs for cancer-specific targeting to selectively and effectively kill cancer cells via amplification of oxidative stress by elevating the level of ROS and reducing the level of GSH.

Herein, an acidic pH-responsive liposome was selected as the nanocarrier because of its biocompatibility and biodegradability to integrate Tf, DHA, and BSO. Furthermore, a fluorescent probe, CellROX, was incorporated for oxidative stress imaging. We designed and synthesized Tf-decorated liposomal nanoparticles with DHA and CellROX encapsulated in the lipid bilayers and BSO in the aqueous core (Tf-DBC NPs) (Scheme 1). Tf-DBC NPs can target the Tf receptor overexpressed on cancer cells and then be uptaken into the lysosomes of cells through a Tf receptor-mediated and clathrin-dependent endocytosis pathway. Under the acidic environment of the lysosomes, the acid-sensing liposome is dissolved to release DHA, CellROX, and BSO. Meanwhile, extra Fe(II) is also generated from Tf which can catalyze DHA to produce abundant ROS. The released BSO can inhibit the synthesis of GSH in cancer cells, thus achieving amplified oxidative stress-mediated cancer cell death. The intracellular oxidative stress can be reflected by the fluorescence of CellROX, providing a visual and convenient protocol to *in situ* monitor the therapeutic efficacy. Therefore, this study offers a new paradigm to achieve amplification of oxidative stress-mediated cancer theranostics.

## Materials and Methods

### Reagents

DHA was purchased from Aladdin Co. Ltd. (Shanghai, China). 1, 2-dioleoylsn-glycero-3-phosphoethanolamine (DOPE), cholesteryl hemisuccinate (CHEMS), BSO and FeSO_4_•7H_2_O were obtained from Sigma-Aldrich (St. Louis, MO, USA). Deferiprone (DEF) was purchased from Meyer Chemical Technology Co. Ltd (Shanghai, China). ROS Detection Kit, Glutathione Assay Kit, Annexin V-FITC/Propidium Iodide (PI) Cell Apoptosis Detection Kit, dihydroethidium (DHE), and Protein Extraction Kit were obtained from KeyGen Biotech. Co. Ltd. (Nanjing, China). BCA Protein Assay Kit was purchased from Beyo-time Institute of Biotechnology (Shanghai, China). The primary antibodies and secondary antibody against TfR and GAPDH were acquired from Affinity Biosciences (Changzhou, China). Fluorescein isothiocyanate (FITC), CellROX, LysoTracker Red, MitoTracker Red, Hoechst 33342, acridine orange (AO) and LIVE/DEAD™ Fixable Green Dead were obtained from Invitrogen (ThermoFisher Scientific, USA). Iron Colorimetric Assay Kit was purchased from BioVision (San Francisco, USA). 1, 2-dipalmitoyl-sn-glycero-3-phosphoethanolamine-*N*-(polyethylene glycol) 2000-transferrin (DSPE-PEG_2000_-Tf) was synthesized by Xi-An Ruixi Biological Technology Co., Ltd. (Xi-An, China). Ultrapure water was prepared using a Millipore Simplicity System (Millipore, Bedford, USA).

### Apparatus

Absorption spectra were recorded on a Cary Series UV-vis and a Cary Eclipse spectrophotometer (Agilent Technologies, USA). The size of the nanodrug was determined by dynamic light scattering (DLS) at 25 °C using a 90 Plus/BI-MAS equipment (Brookhaven, USA). The morphology of the nanodrug was characterized at a JEOL JEM-200CX transmission electron microscope (TEM) operated at 200 kV. Zeta potential measurement was performed at 25 °C on a Zetasizer (Nano-Z, Malvern, UK). Confocal fluorescence imaging of cells was performed on a confocal laser scanning microscope (CLSM, LSM800, Zeiss, Germany). Flow cytometric assay was performed using MACSQuant Analyzer 10 (MiltenyiBiotec, Germany). *In vivo* fluorescence imaging experiments were performed on a Maestro EX *in vivo* imaging system (CRI, Inc.). The hematoxylin and eosin (H&E) staining images and TUNEL staining images were acquired on a digital pathology slice scanner using NanoZoomer 2.0 RS (Hamamatsu, China). The immunoreactive bands of Western Blot were visualized by the ChemiDoc™ MP System (Bio-Rad, Hercules, CA, USA) and analyzed using the ImageLab™ software.

### Synthesis of Tf-DBC NPs

Tf-DBC NPs were prepared by a thin-film hydration method. In brief, a mixture of DSPE-PEG_2000_-Tf, DOPE, and CHEMS at a molar ratio of 0.5:6:4 were used for the liposome formulation. 10 mg DHA and 1 mmol CellROX were dissolved in 2 mL solvent composed of chloroform: methanol (2:1, v/v). The solution was evaporated to dryness at 50 °C for several minutes until the formation of the thin lipid film at the bottom. Subsequently, the lipid film in the bottle was redissolved using 10 mL sterile phosphate buffered saline (PBS) containing 5 mg BSO. To prepare the well-dispersed NPs, the solution was subjected to ultrasonic processing and filtered with a 0.22 μm polycarbonate membrane.

### Characterization of Tf-DBC NPs

The Tf-DBC NPs were characterized in terms of morphology, particle size, surface charge, and encapsulation efficiency. The solution of Tf-DBC NPs was dropped onto a carbon-coated copper grid and then stained with 2.0% (w/v) phosphotungstic acid for TEM measurement. The encapsulation efficiency of DHA and BSO were determined using the HPLC method with the detection wavelength of 215 nm and 335 nm, respectively. All determinations were performed at least three times.

### Encapsulation Efficiency and Loading Capacity of Tf-DBC NPs

The encapsulation efficiency (EE) and loading capacity (LC) determination of Tf-DBC NPs were performed by a dialysis method. Tf-DBC NPs were placed in 30 kDa dialysis chambers, dialyzed in 50 mL of pH 7.4 citrate buffer, and 200 μL of the withdrawn sample was dried and dissolved in 95% ethyl alcohol (200 μL). Next, 800 μL of 0.2% NaOH solution was added and incubated for 30 min at 50 °C. The supernatant was obtained by centrifugation at 14000 rpm for 10 min and assayed by HPLC for DHA and BSO. The concentration of CellROX was measured by the fluorescent method. The EE of content was calculated as EE (%) = (*W*_1_-*W*_2_)/*W*_1_×100%, where *W*_1_ and *W*_2_ were the weights of added content and unloaded content, respectively. The LC of content was calculated as LC (%) = (*W*_1_-*W*_2_)/*W*_t_×100%, where *W*_1_ and *W*_2_ were the weights of added content and unloaded content, respectively, and *W*_t_ was the complete weight of nanoparticles.

### Cell Culture

The hepatocarcinoma HepG2 cell line and human normal liver L-02 cell line were obtained from KeyGen Biotech Co. Ltd. (Nanjing, China). All cell lines were cultured in Dulbecco's modified Eagle's medium (DMEM) containing 10% FBS, 100 μg mL^-1^ streptomycin and 100 U mL^-1^ penicillin at 37 °C in a humidified incubator containing 5% CO_2_ and 95% air. The medium was replenished every other day, and the cells were sub-cultured after reaching confluence.

### Confocal Fluorescence Imaging for Living Cells

HepG2 cells and L-02 cells were seeded in 35-mm glass-bottom confocal dishes at a density of 1 × 10^4^ per dish and incubated with Tf-DBC NPs (2.5 μM DHA equiv.) or Tf-FITC NPs (5.0 μM FITC equiv.) at 37 °C for different times. After incubation, the cells were stained with LysoTracker Red, MitoTracker Red, Hoechst 33342, AO and LIVE/DEAD. Cell imaging was then carried out after washing cells three times with PBS to remove any liposomes from the surface of cells. The fluorescence of cells was visualized with a confocal laser scanning microscope with a 63 × oil immersion objective lens using fixed parameters including the laser intensity and exposure time. The fluorescence signal of Hoechst 33342 was excited with a 405 nm helium-neon laser and emission was collected from 420 to 480 nm. The fluorescence signal of cells incubated with FITC was excited with a 488 nm helium-neon laser and emission was collected from 505 to 540 nm. LysoTracker Red and MitoTracker Red were both excited at 543 nm with an argon ion laser, and the emission was collected from 570 to 620 nm. CellROX was excited at 635 nm with an argon ion laser, and the emission was collected from 650 to 720 nm.

### Cytotoxicity Assay

To investigate the cytotoxicity of Tf-DBC NPs, standard methyl thiazolyl tetrazolium (MTT) tests were performed on HepG2 cells. Briefly, HepG2 cells were seeded in a 96-well plate at a density of 3 × 10^3^ cells per well in 100 μL complete medium and incubated at 37 °C for 24 h. After rinsing with PBS, HepG2 cells were incubated with 100 μL culture media containing serial concentrations of Tf-DBC NPs, DHA, BSO and free drugs (DHA + BSO) for 24 h. Subsequently, 20 μL of MTT (5 mg mL^-1^ in PBS) was added to each well followed by incubation for 4 h. Next, the supernatants containing unreacted MTT were discarded, and 150 μL DMSO was added to each well to dissolve the produced blue formazan. The absorbance was recorded at 550 nm using a microplate reader after 10 min of shaking. The cell viability was then calculated as follows: Cell viability (%) = [(OD value of treatment group - OD value of blank group)/(OD value of control group - OD value of blank group)] × 100%.

### Detection of Fe(II) and GSH

HepG2 and L-02 cells were separately seeded in 6-well plates and cultured in 2 mL of medium for 24 h. Subsequently, Tf NPs (Tf decorated liposomal nanoparticles) were added while the incomplete medium was used for control. After incubation for 8 h, the cells were collected and the concentration of Fe(II) analyzed by Iron Colorimetric Assay Kit according to the manufacturer's instructions. The total GSH contents in HepG2 cells were measured by Glutathione Assay Kit. The GSH content in HepG2 cells was calculated according to the formula for tissue: Content of GSH (mg /g prot) = [(absorbance value of sample - absorbance value of blank)/(absorbance value of standard - absorbance value of blank)] × concentration of standard (20 µmol /L) × molecular weight of GSH (307 g /mol) ÷ total protein concentration of sample (g prot /L).

### Flow Cytometric Assay

HepG2 cells were seeded in 6-well plates (1 × 10^5^ per well) and incubated in complete medium for 24 h at 37 °C and were randomly divided into the following nine groups and treated as follows: control; DBC NPs (liposomal nanoparticles loaded with DHA, BSO, and CellROX); Tf NPs; Tf-DBC NPs (Tf-decorated liposomal nanoparticles load with DHA, BSO, and CellROX); Apo-Tf-DBC NPs group (apotransferrin-decorated NPs loaded with DHA, BSO, and CellROX); Tf + Tf-DBC NPs group (free Tf was added to HepG2 cells for 30 min before incubation with Tf-DBC NPs); free drugs (free DHA and BSO); Tf-DC NPs group (Tf-decorated liposomal nanoparticles loaded with DHA and CellROX); Tf-BC NPs (Tf-decorated liposomal nanoparticles loaded with BSO and CellROX). After 24 h treatment, the nine groups of HepG2 were stained with Annexin V-FITC and PI, trypsinized, harvested, rinsed with PBS, resuspended, and subjected to flow cytometric assay. All experiments detected at least 10,000 cells and the data were analyzed with the Flowjo software (TreeStar Inc).

### Western Blot Analysis

5×10^7^ HepG2, L-02, HK-2, HUVEC, and H9c2 cells were collected after washing with the ice-cold PBS and the cell membrane proteins were obtained following the operation manual instructions of the protein extraction kit. Subsequently, the protein concentration was calculated using a BCA protein assay kit. Equal amounts of proteins (30 µg) were separated on a 12.5% SDS-PAGE and were transferred to PVDF membrane by electroblotting. Membranes were incubated overnight at 4 °C with the appropriate primary antibodies against TfR and GAPDH (dilution 1:1000). The membranes were then probed with the secondary antibody at a 1:3000 dilution. The antigen-antibody complexes were then detected with enhanced chemiluminescence (ECL) reagent. Finally, the immunoreactive bands were visualized by the ChemiDoc™ MP Systemand analyzed using the Image Lab™ software.

### Animals and Tumor Model

Specific pathogen-free female BALB/c nude mice, 5-6 weeks of age, were purchased from Shanghai Laboratory Animal Center, Chinese Academy of Sciences (SLACCAS) and bred in an axenic environment. All animal operations were in accord with institutional animal use and care regulations approved by the Model Animal Research Center of China Pharmaceutical University. HepG2 tumor model was established by subcutaneous injection of HepG2 cells (1 × 10^6^) into the armpit of the nude mice with further culture to obtain the tumors grown to 4-5 mm in diameter for *in vivo* fluorescence imaging.

### *In Vivo* Study of Antitumor Efficacy

For investigating the antitumor effect *in vivo*, HepG2 tumor-bearing mice were subjected to seven different treatments through tail vein injections in the following groups: saline control; Tf-DBC NPs; Tf-DC NPs; Tf-BC NPs; Apo-Tf-DBC NPs; DBC NPs and free DHA and BSO drugs. A single dose of each formulation was administrated into the tail vein in 200 μL at a dose of 0.9 mg kg^-1^ DHA equivalent every day for 15 days. The tumor volume was calculated using the length × width^2^ × 0.5 determined by a vernier caliper. During the experiment, mice were anesthetized with 2.5% isoflurane in oxygen delivered at a flow rate of 1.5 L min^-1^. The body weight of mice was also recorded every day. Major organs including heart, liver, spleen, lung, kidney, and tumor were excised, followed by washing the surface of each tissue with physiological saline several times for *ex vivo* imaging and semiquantitative analyses. The tissues were embedded in paraffin and cut at 5 μm thickness. The tumor and the organs sections were stained with hematoxylin and eosin (H&E) and TUNEL for histopathological evaluation.

### *In Vivo* Fluorescence Imaging

To evaluate the targeting ability of Tf and monitor the generation of ROS in tumor cells, we administered BALB/c nude mice bearing HepG2 tumors with Tf-DBC NPs, Apo Tf-DBC NPs, DBC NPs, Tf-DC NPs, and Tf-BC NPs at a dose corresponding to 0.9 mg kg^-1^ of DHA each into the tail vein. The mice were anesthetized by isoflurane for fluorescence imaging at the monitoring times (0, 2, 4, 8, and 12 h). Fluorescence signals were detected by *in vivo* fluorescence imaging system with excitation at 635 nm and emission from 650 to 700 nm.

### Statistical Analysis

Data were expressed as means ± SD from at least three experiments. Statistical analyses were carried out using a statistics program (GraphPad Prism; GraphPad Software). One-way ANOVA was used to compare the treatment effects. *P<*0.05 was considered to be statistically significant.

## Results and Discussion

### Characterization of Tf-DBC NPs

**Figure 1:**
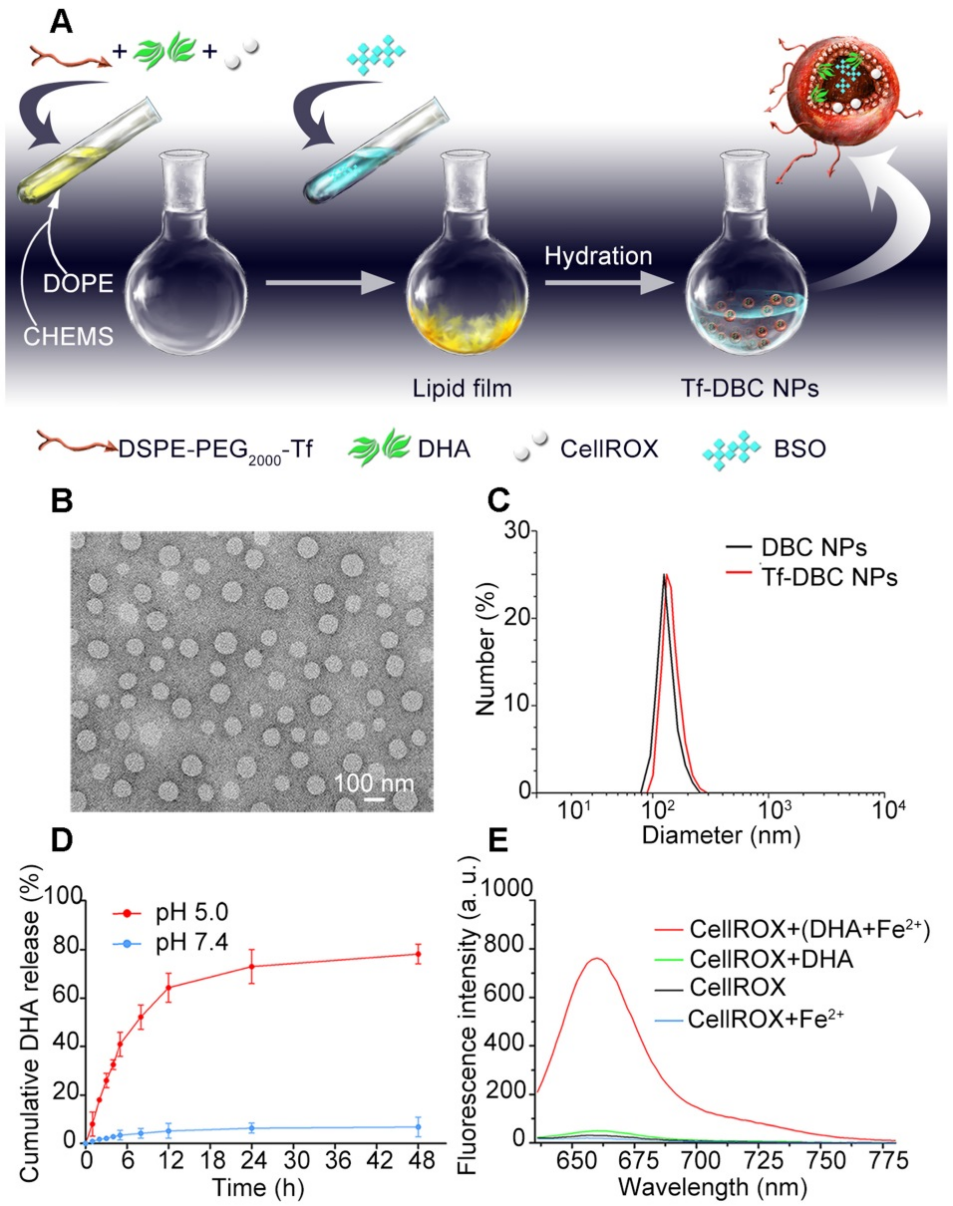
(A) Synthesis of Tf-DBC NPs; (B) TEM images of Tf-DBC NPs; (C) Hydrodynamic diameters of Tf-DBC NPs and DBC NPs; (D) Release of DHA from Tf-DBC NPs at pH 7.4 and 5.0; (E) Fluorescence response of CellROX at 640 nm in the presence of DHA and Fe(II), DHA only, and Fe(II) only.

Liposomes are spherical vesicles with a bilayer membrane structure composed of phospholipids and have been widely used for drug delivery because of their versatile loading capacities for various types of drugs and excellent biocompatibility 39. Importantly, liposomes can be designed to be pH-responsive by controlling their compositions 40. In this study, liposomes were selected as the nanocarrier and assembled by utilizing DSPE-PEG_2000_-Tf, DOPE, and CHEMS. The hydrophobic DHA and CellROX were added during the formation of the lipid membrane, whereas the hydrophilic BSO was encapsulated in the aqueous core. The Tf-decorated and DHA, BSO, and CellROX co-loaded liposomal nanoparticles (Tf-DBC NPs) were prepared by a thin-film hydration method (Figure 1A) and then characterized in terms of morphology, particle size, and surface charge. As was evident by TEM and DLS analyses, Tf-DBC NPs displayed spherical shapes with nanosized structure and dispersed uniformly (Figure 1B). The hydrodynamic diameters of DBC NPs and Tf-DBC NPs were 118.4 ± 3.4 nm and 127.2 ± 5.8 nm, respectively (Figure 1C). The slight increase in the size of Tf-DBC NPs could be ascribed to the Tf decoration on the liposomes. Moreover, there was a strong characteristic absorption peak at 280 nm (Figure S1) following the decoration of liposomes with Tf compared with the blank liposomes, demonstrating the successful Tf grafting on the lipid bilayers. The zeta potential of DBC NPs and Tf-DBC NPs were -22.3 ± 0.18 and -27.1 ± 0.16 mV, respectively, indicating that the embedded Tf did not influence the stability of the liposomes 41. The PDI values of Tf-DBC NPs and DBC NPs were also almost the same (Table S1). The loading capacity of DHA and BSO were 17.9% and 4.1% and the encapsulation efficiency was determined to be 76.4% and 33.3% (Table S2) through HPLC method with the detection wavelength of 215 nm and 335 nm, respectively (Figure S2 and S3).

To evaluate the stability of the NPs, the size measurement of hydrodynamic diameter was performed under different conditions. The Tf-DBC NPs maintained size stability in serum and PBS for at least 7 days (Figure S4). The HPLC method was also used to determine the accumulative release percentage of DHA and BSO (Figure 1D and S5) and was found to be below 10% at pH 7.4 after 24 h incubation. However, when the pH decreased to 5.0, the accumulative release percentage of DHA and BSO reached 79.46% and 82.54%, respectively, after 24 h incubation, demonstrating the pH-responsive drug release property of Tf-DBC NPs.

### Fluorescence Response of CellROX

CellROX is a commercial fluorescence probe for measuring cellular oxidative stress in both live and fixed cell imaging 42. This fluorescent dye is non-fluorescent in a reduced state, and exhibits bright fluorescence upon oxidation by ROS. As shown in Figure S6, in the presence of ROS, the fluorescence spectra of CellROX consisted of the optimal excitation and emission wavelengths of 640 nm and 665 nm, respectively. Furthermore, in the presence of both Fe(II) and DHA, there was a strong fluorescent signal of CellROX. In contrast, when there was only DHA or Fe(II) present, the fluorescent signal of CellROX was low (Figure 1E), demonstrating that Fe(II) was the critical element to induce the production of ROS from DHA. Also, the fluorescence response of CellROX encapsulated in the complete Tf-DBC NPs in the acidic environment at pH 5.0 by ferri reductase was also evaluated (Figure S7), which verified that the complete Tf-DBC NPs could be induced to generate ROS with the property of self-monitoring the oxidative stress.

### Evaluation of Tf Function

**Figure 2:**
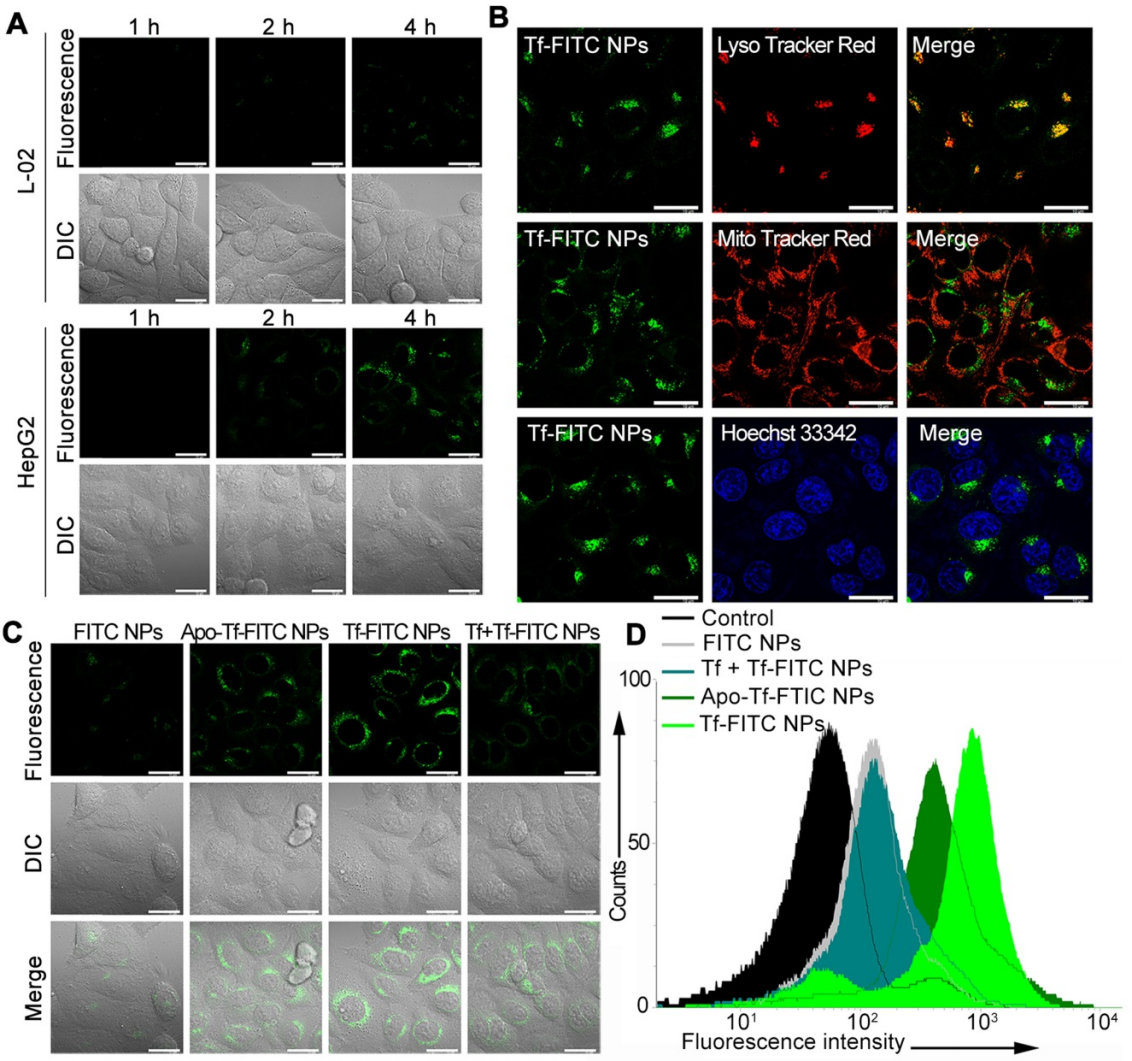
(A) Confocal fluorescence images of HepG2 and L-02 cells incubated with Tf-FITC NPs for different times. Scale bars: 10 μm. (B) Confocal fluorescence images of HepG2 cells stained with LysoTracker Red, Mito Tracker Red and Hoechst 33342 following incubation with Tf-FITC NPs for 4 h. Scale bars: 10 μm. (C) Confocal fluorescence images of HepG2 cells with different treatments as indicated. Scale bars: 10 μm. (D) Flow cytometric assay for the targeting ability of Tf and iron ions with FITC NPs, Apo-Tf-FITC (preprocessed by deferrization), Tf-FITC NPs, and Tf + Tf-FITC NPs (preincubated with Tf).

To study the targeting capability of the nanocarrier, Tf-decorated and FITC-loaded liposomal nanoparticles (Tf-FITC NPs) were prepared. After incubating HepG2 and L-02 cells with Tf-FITC NPs, green fluorescent spots were observed within 2 h in HepG2 cells; the intensity of fluorescence increased with increasing incubation time (Figure 2A), indicating rapid internalization of Tf-FITC NPs into the cancer cells. In contrast, L-02 cells showed a faint fluorescent signal even after incubation with Tf-FITC NPs for 4 h. The pixel intensity of FITC was calculated in HepG2 and L-02 cells with different incubation times (Figure S8), which further demonstrated that Tf-FITC NPs could be effectively internalized by cancer cells. The confocal fluorescence images of HepG2 cells co-stained with LysoTracker Red as a lysosomal indicator, MitoTracker Red as a mitochondrial indicator, and Hoechst 33342 as a nuclear indicator were displayed in Figure 2B. The yellow fluorescence spots originating from the fluorescence overlapping of FITC dye and LysoTracker Red were observed indicating that the accumulation and localization of Tf-FITC NPs were in the lysosomal compartments (Figure 2B).

TfR is a homodimeric type II membrane protein that plays a critical role in the iron acquisition mechanism for all iron-requiring cell types 35 and the iron ion was determined to be necessary for the targeting capability of Tf. To determine whether Tf enhances the targeting ability of nanoparticles to cancer cells, HepG2 cells were incubated with FITC-loaded NPs without Tf decoration (FITC NPs) and apo-transferrin decorated NPs (Apo-Tf-FITC NPs). The green fluorescence in cells treated with FITC NPs was much weaker compared with those treated with the Tf-FITC NPs and Apo-Tf-FITC NPs (Figure 2C). The intracellular fluorescence intensity treated with Tf-FITC NPs was higher than that treated with Apo-Tf-FITC. As an additional control, the free Tf was added to HepG2 cells 30 min before incubation with Tf-FITC NPs (Tf + Tf-FITC NPs). The fluorescence intensity decreased significantly compared with Tf-FITC NPs group, which indicated that Tf played an important role in the targeted delivery of NPs into cancer cells. The flow cytometry (Figure 2D) assays of different groups also verified the important role of Tf in the targeted delivery of Tf-FITC NPs to the Tf receptor over-expressing cancer cells.

**Figure 3:**
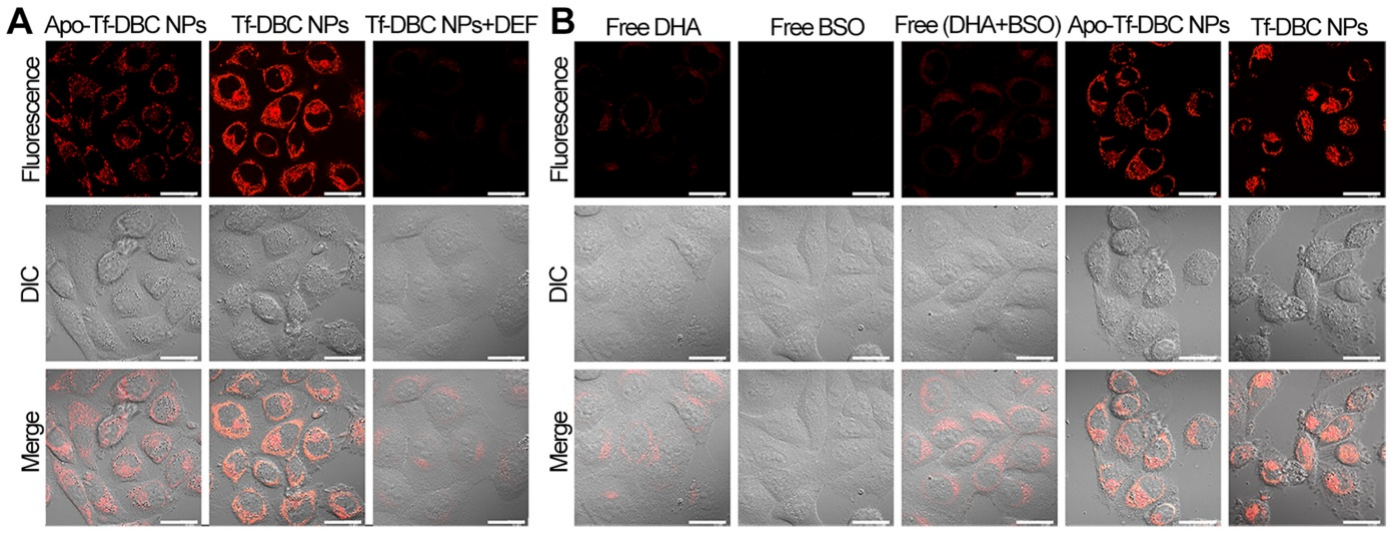
(A) Confocal fluorescence images of HepG2 cells treated with Apo-Tf-DBC NPs, Tf-DBC NPs, and Tf-DBC NPs in the presence of deferiprone DEF as a scavenger of Fe(II). (B) Confocal fluorescence images of HepG2 cells incubated with free DHA, free BSO, free DHA, and BSO, Apo-Tf-DBC NPs, and Tf-DBC NPs. Scale bars: 10 μm.

To analyze the generation of ROS by the Tf-DBC NPs, HepG2 cells were incubated with Tf-DBC NPs. Red fluorescent spots were observed in HepG2 cells in 1 h the intensity of which increased with increasing incubation time (Figure S9). Bright fluorescence was observed after 4 h incubation and the cells showed apoptosis in 6 h, indicating the gradual increase in the generation of ROS in HepG2 cells; hence, the best incubation time of Tf-DBC NPs was determined to be 4 h. To determine whether Tf enhances the generation of ROS through cleavage of the endoperoxide bridge of DHA by supplying Fe(II), HepG2 cells were incubated with Apo-Tf-DBC NPs in which Tf was preprocessed by deferrization. When the fluorescent intensity of Apo-Tf-DBC NPs and Tf-DBC NPs were compared, bright fluorescence was exclusively observed in HepG2 cells treated with Tf-DBC NPs (Figure 3A) demonstrating an essential role of iron ion during the generation of ROS. Furthermore, considerably decreased fluorescence intensity was observed in HepG2 cells treated with Tf-DBC NPs and deferiprone (DEF) as a scavenger of Fe(II), indicating the requirement of Fe(II) for Tf-DBC NPs-induced ROS generation in cancer cells. The flow cytometric analysis also demonstrated that the iron ion was essential for the generation of ROS (Figure S10A). To further evaluate the role of ROS generated by Tf and DHA in inducing cell apoptosis, the MTT assay was used to evaluate the cytotoxicity of HepG2 cells treated with Tf-DBC NPs, Apo-Tf-DBC NPs, and Tf-DBC NPs in the presence of deferiprone DEF as a scavenger of Fe(II). The results presented in Figure S10B confirmed that Tf played an important role in cell apoptosis.

We analyzed the fluorescence signals of CellROX in HepG2cells treated with free DHA and/or BSO, Tf-DBC, or Apo-Tf-DBC NPs. CellROX did not emit fluorescence when the cells were incubated with free DHA and/or BSO. However, upon treatment of HepG2 cells with Tf-DBC NPs, CellROX bound to the cells and emitted intense fluorescence, which demonstrated that the overproduction of ROS was due to the Tf-DBC NPs (Figure 3B). Bright field imaging analysis showed no changes in the morphology of HepG2 cells in the free drug groups (DHA and/or BSO), whereas cells incubated with Tf-DBC NPs underwent cell death. As for cell viability, with 30 µM DHA, almost 60% of the cells survived in the free DHA and/or BSO groups while Tf-DBC NPs were effective in killing HepG2 cells with 50% growth inhibition concentration (IC_50_) of 2.5 μM (DHA equiv.) (Figure S11). Thus, the cumulative results of fluorescence emission, morphology changes, and cell viability of HepG2 cells in the presence of Tf-DBC NPs offered direct evidence for the dual functions of Tf as the targeting moiety for Tf receptors overexpressed on tumor cells and as a ferric ion carrier for providing Fe(II) to catalyze DHA and generate abundant ROS. These results indicated that Tf-DBC NPs with the self-feedback fluorescence of intracellular ROS provided a reliable and convenient method for monitoring of ROS-mediated cell death.

### Anticancer Activity of Tf-DBC NPs via Amplification of Oxidative Stress

The intracellular iron ion and GSH were essential factors for the amplification of oxidative stress to kill cancer cells. The iron ion content was detected by an iron colorimetric assay kit. The comparison of the iron ions including Fe(II) and Fe(III) between HepG2 and L-02 cells showed that the iron ion content in HepG2 cells was much higher than that in L-02 cells (Figure S12). After cells were incubated with Tf-DBC NPs, the iron ion concentration remarkably increased in HepG2 cells while no significant change was observed in L-02 cells. This could be attributed to the fact that Tf receptors were overexpressed on the membranes of HepG2 cells and, therefore, the uptake of Tf-DBC NPs by HepG2 was much higher than that by L-02 cells. This result again confirmed the selectivity of Tf for cancer cells.

We examined the level of the antioxidant GSH in HepG2 cells to verify its depletion by BSO. A high level of GSH was present in the control group, while Tf-DBC NP-treated group had a significantly reduced GSH level (Figure S13) suggesting that BSO encapsulated in the Tf-DBC NPs eliminated the cellular GSH. Though the level of GSH was also decreased in the free BSO group, it was still much higher than that in the groups treated the Tf-B NPs and Tf-DBC NPs. This result also showed that Tf-DBC could be readily uptaken by HepG2 cancer cells leading to rapid and remarkable reduction in the GSH level.

To investigate whether the generated ROS affected cell viability, the LIVE/DEAD detection assay was employed. In cells with compromised membranes, the dye reacts with free amines both in the cell interior and on the cell surface, yielding intense fluorescent staining. In viable cells, on the other hand, the dye's reactivity is restricted to the cell-surface amines, resulting in less intense fluorescence 43. When HepG2 cells were treated with Tf-C NPs and Tf-BC NPs, only a little red fluorescence of CellROX and green fluorescence of cell viability assay were observed on the cell surface. For the HepG2 cells treated with Tf-DBC NPs and Tf-DC NPs, the red fluorescence from CellROX and green fluorescence from LIVE/DEAD were remarkably increased, demonstrating that Tf-DBC NPs and Tf-DC NPs could produce abundant ROS which induced cell apoptosis with Tf-DBC NPs inducing a higher fluorescence than Tf-DC (Figure S14). Thus, integrating Tf, DHA, and BSO into one nanoparticle could effectively kill cancer cells via the amplification of oxidative stress strategy. The pixel intensity of CellROX and LIVE/DEAD fluorescence calculated in HepG2 cells after incubating with Tf-C NPs, Tf-BC NPs, Tf-DC NPs, and Tf-DBC NPs was also shown in Figure S15, which further demonstrated that Tf-DBC NPs could produce abundant ROS to effectively kill tumor cells and also allow self-monitoring.

The accumulation of Tf-DBC NPs in the lysosomal compartment has been reported to generate ROS, which might trigger lysosomal membrane permeabilization (LMP)-associated cell death pathway 4. To further investigate whether the generated ROS affected the integrity of lysosomes, AO was employed as an indicator. HepG2 cells were incubated with different groups of nanoparticles to compare the red fluorescence of AO, which stained intact lysosomes. As was evident from Figure S16, the red fluorescence from AO was remarkably decreased when HepG2 cells were incubated with Tf-DBC NPs, demonstrating that ROS generated by Tf-DBC NPs could cause LMP to induce cell apoptosis.

**Figure 4:**
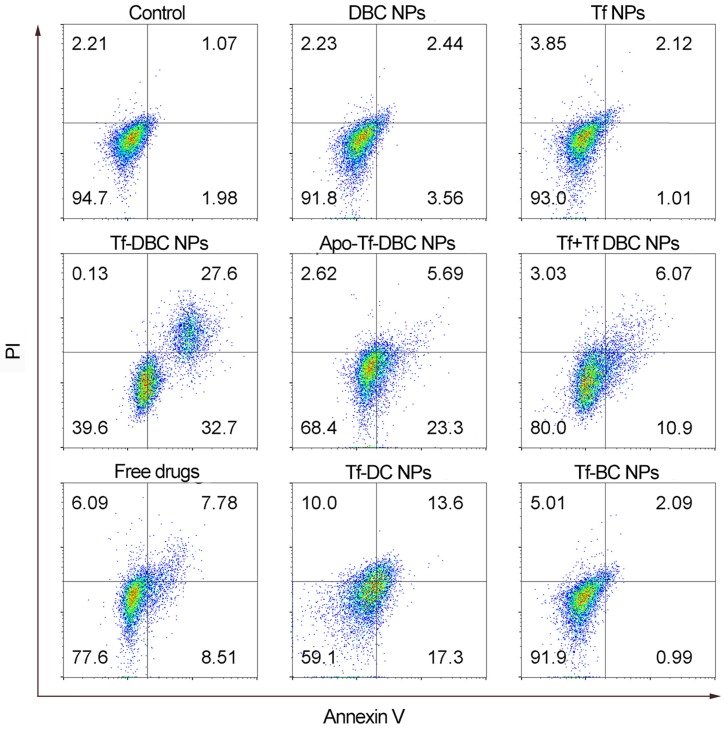
Flow cytometric apoptotic analysis of HepG2 cells induced by different formulations.

To better explore the cytotoxicity of DBC NPs with the self-amplification of oxidative stress, the drug-induced cell death was examined by flow cytometry. As shown in Figure 4, HepG2 cells treated with Tf-DBC NPs exhibited cell mortality of 60.3% which was significantly higher than that of the free DHA and BSO groups with the rate of only 15.15%. The cell mortality was reduced significantly to 16.97% for the group with Tf block blockage and 28.99% for the Apo-Tf-DBC NPs group, clearly suggesting that Tf performed its dual functions of targeting the receptors on cancer cells as well as inducing apoptosis. The co-loading of DHA and BSO in the nanoparticles resulted in synergistic cytotoxicity compared with individual loading in the liposomes. The percentage of cell apoptosis treated with Tf-DC NPs and Tf-BC NPs were 30.6% and 3.08%, respectively. Thus, the amplified oxidative stress in tumor cells could effectively induce cancer cell death.

### Cytotoxic Selectivity of Tf-DBC NPs

The Tf-mediated targeted cell death was investigated by incubating Tf-DBC NPs with HepG2 cells that highly express Tf receptors, and L-02, H9c2, HK-2, and HUVEC cells with low expression of these receptors. Western blotting confirmed noticeably higher expression of TfR on HepG2 cells compared with L-02, H9c2, HK-2, and HUVEC cells (Figures S17A and S17B). Also, the fluorescent signal from CellROX was obvious in HepG2 cells while almost no signal was detected in all four cell lines with low TfR expression (Figure S18A). Moreover, the MTT assay further verified the cytotoxic selectivity of Tf-DBC NPs. Unlike HepG2 cells, the cell viability of L-02, H9c2, HK-2, and HUVEC cells remained above 80% following incubation with Tf-DBC NPs (Figure S18B). All these results demonstrated the therapeutic selectivity of Tf-DBC NPs for TfR-expressing cancer cells which would minimize the side-effects to normal cells.

### *In vivo* antitumor effect of Tf-DBC NPs

To illustrate the blood circulation profile of the nanodrug, we prepared Tf-decorated liposomal nanoparticles encapsulating Cyanine7 (Tf-Cy7 NPs). The time profile of plasma concentration of Tf-Cy7 NPs was illustrated in Figure S19 with a rapid clearance during the first 4 h followed by a continuous and slow elimination rate. According to pharmacokinetics analysis, the two-compartment model was most fitted to describe the pharmacokinetics of Tf-Cy7 NPs after intravenous (i.v.) administration. The blood circulation half-life was 4.81 h, which indicated a long persistence of Tf-Cy7 NPs in the bloodstream to corroborate the stability *in vivo*. The whole-body fluorescence images at different time points in the biodistribution study of Tf-Cy7 NPs were also evaluated (Figure S20). After i.v. injection of Tf-Cy7 NPs into HepG2 tumor-bearing mice, the tumor site could be distinguished from the surrounding normal tissue at 2 h post-injection. The fluorescence of Cy7 increased gradually and reached a maximum at 12 h post-injection demonstrating the targeted delivery of Tf-Cy7 in the tumor tissue. The *ex-vivo* images of the tumor and other organs were also evaluated (Figure S21), which confirmed the tumor-targeting capability of the Tf-Cy7 NPs.

**Figure 5:**
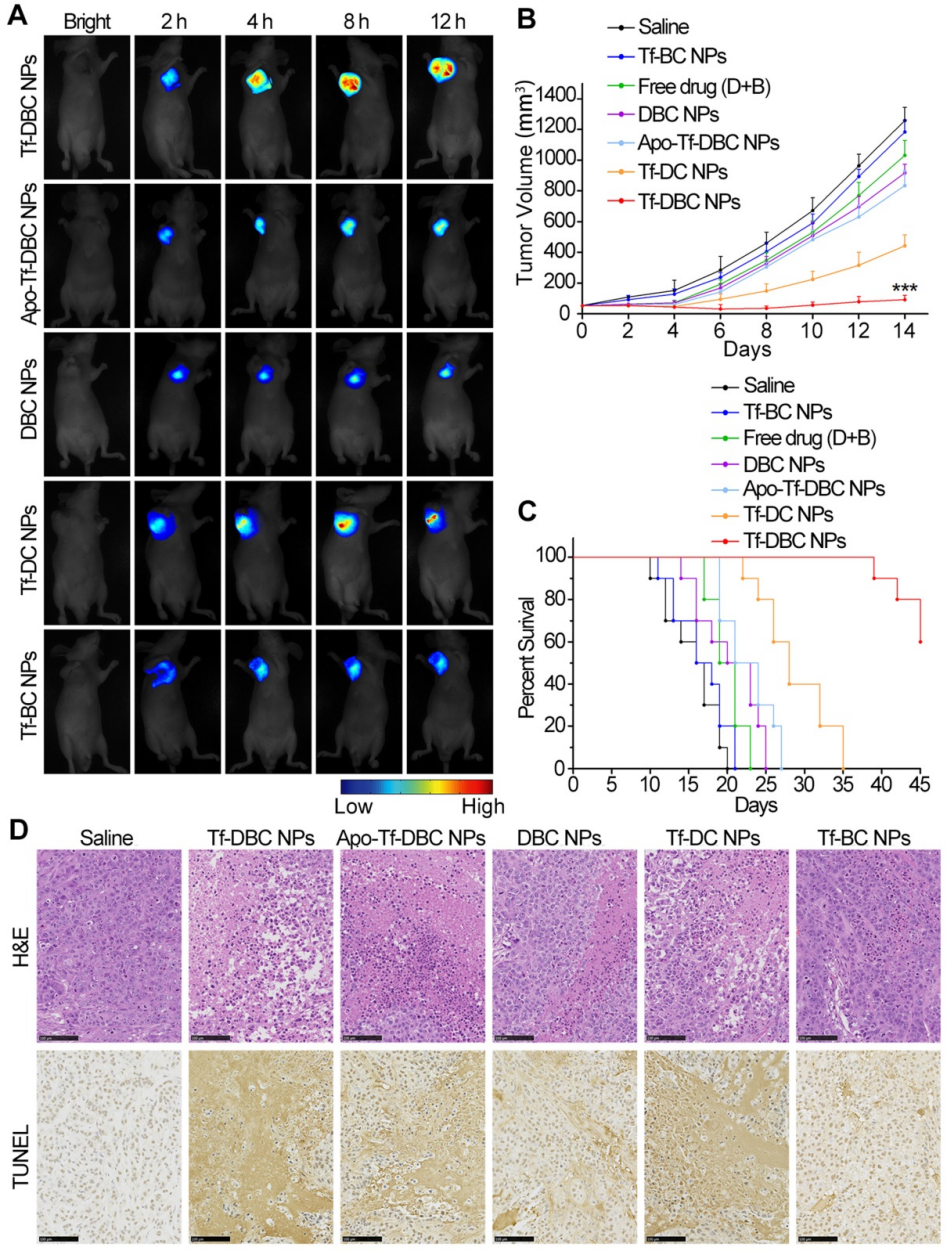
(A) *In vivo* time-dependent fluorescence imaging of HepG2 tumor-bearing nude mice after intravenous injection with Tf-DBC NPs, Apo-Tf-DBC NPs, DBC NPs, Tf-DC NPs, and Tf-BC NPs. (B) Measurement of tumor volume growth after treatment with different formulations (n=6). (C) Survival rates of HepG2 tumor-bearing mice after intravenous injection with different formulations within 45 days. (D) Representative H&E-stained and TUNEL-stained histological sections of tumor tissue after treatment with different formulations. Scale bars: 100 μm.

Next, to examine the targeting and monitoring ability of Tf-DBC NPs in the *in vivo* tumors, the mice bearing subcutaneously implanted HepG2 tumors were administered with intravenous injection via tail vein of Tf-DBC NPs at the dosage equivalent to 0.9 mg kg^-1^ of DHA and scanned at different time points after injection. The fluorescent signal intensity of the probe increased with increasing time from 2 h and was observed exclusively in the tumor region without background signal during 4 to 12 h post-injection of Tf-DBC NPs (Figure 5A), confirming that Tf-DBC NPs could accumulate in the tumor and effectively generate ROS.

To further verify that the fluorescent signal originated from the accumulated probe in the tumor, we recorded *ex-vivo* images of the tumor and other organs collected immediately after killing the mice at 12 h post-injection. Consistent with the results observed *in vivo*, only the tumor yielded a strong fluorescent signal (Figure S22) after injection of Tf-DBC NPs. The semiquantitative biodistribution of Tf-DBC NPs in liver, heart, lung, kidney, spleen, and tumor in HepG2 tumor-bearing mice was also determined (Figure S23), which demonstrated that Tf-DBC NPs could effectively accumulate in the tumor and generate abundant ROS. The targeting capability and the catalytic function of Tf to generate ROS was confirmed by injecting Apo-Tf-DBC NPs and DBC NPs into the mice that showed a faint fluorescence signal in the tumor region (Figure 5A). The emergence and increase of the signal after injection of Tf-DBC NPs revealed abundant production of ROS in the tumor by the targeting and catalytic functions of Tf. Comparison of the normalized fluorescence intensities of intratumoral ROS after various treatments of Apo-Tf-DBC NPs, DBC NPs, Tf DBC NPs, Tf-DC NPs, and Tf-BC NPs (Figure S24) revealed that Tf-DBC NPs produced the synergistic effect of the production of abundant ROS and its accumulation in the tumor.

The synergistic effect of Tf-DBC NPs by elevating the oxidative stress and GSH depletion in tumor tissues was expected to suppress the tumor growth efficiently. The long term *in vivo* antitumor efficacy was assessed by measuring the tumor growth in mice treated with Tf-DBC NPs. Tumor growth was significantly suppressed (Figure 5B), which could be attributed to the amplification of oxidative stress. Furthermore, the significant difference in the growth rates of tumors treated with Tf-DBC NPs, Tf-DC NPs, and Tf-BC NPs had demonstrated that Tf-DBC NPs achieved a synergistic effect to suppress tumor growth, indicating that the integration of Tf, DHA and BSO could produce amplification of oxidative stress. Tf-DBC NPs-treated mice showed significantly prolonged survival compared to other groups (Figure 5C), confirming the impact of the amplification of oxidative stress. The mice did not show noticeable body weight loss during 14 days after different treatments (Figure S25). Furthermore, H&E-stained and TUNEL-stained images revealed that tumor tissues in Tf-DBC NPs therapy group suffered more severe damage than those in saline, Tf-DC NPs, Tf-BC NPs and DBC NPs groups (Figure 5D). The potential toxicity of Tf-DBC NPs for organ tissues was also assayed through H&E staining (Figure S26). No noticeable sign of organ damage for lung, liver, spleen, kidney, and heart was observed in the Tf-DBC NPs group and controls, suggesting the negligible side effects of Tf-DBC NPs for *in vivo* cancer treatment. Taken together, the results from the *in vitro* and *in vivo* experiments strongly suggest that Tf-DBC NPs display not only an effective targeting ability but also self-amplification of oxidative stress to kill cancer cells.

## Conclusion

We developed a Tf decorated, DHA and BSO-loaded theranostic nanodrug, which induced cancer cell death by tumor-specific amplification of intracellular oxidative stress. Tf-decorated nanodrug could not only preferentially recognize cancer cells but also supply iron as a catalyst inducing DHA to produce abundant ROS. Furthermore, BSO, by inhibiting GSH synthesis, could decrease the adaptive antioxidants, thus eliciting a high level of oxidative stress in cancer cells. In addition, CellROX, as a fluorescent probe for imaging the intracellular ROS, provided self-feedback for monitoring the process of oxidative stress amplification-mediated cancer therapy. These capabilities highlight the potential of the nanodrug as a valuable theranostic agent for accurate and effective therapeutic outcomes, and also provide a new paradigm to achieve a complete tumor cure using natural products.

## Abbreviations

ROS: reactive oxygen species
DOPE: 1, 2-dioleoylsn-glycero-3-phosphoethanolamine
CHEMS: cholesteryl hemisuccinate
FITC: Fluorescein isothiocyanate
DSPE-PEG_2000_-Tf: 1, 2-dipalmitoyl-sn-glycero-3-phosphoethanolamine-*N*-(polyethylene glycol)_2000_-Transferrin
TEM: transmission electron microscopy
DLS: dynamic light scattering
HPLC: high performance liquid chromatography
MTT: methyl thiazolyl tetrazolium
