# Supplementary material for: A cancer-specific activatable theranostic nanodrug for enhanced therapeutic efficacy via amplification of oxidative stress
Source: Theranostics. 2020 Jan 1;10(1):371–83. doi: 10.7150/thno.39412 (PMC6929611; doi:10.7150/thno.39412)
Supplement: Supplementary file 1 — Supplementary figures and tables. [file thnov10p0371s1.pdf]

Supporting Information

A cancer-specific activatable theranostic nanodrug for enhanced therapeutic efficacy via amplification of oxidative stress

Xie-an Yu*, Mi Lu*, Yingping Luo, Yiting Hu, Ying Zhang, Zhiming Xu, Shuaishuai Gong, Yunhao Wu, Xiao-Nan Ma, Bo-Yang Yu✉, Jiangwei Tian✉

State Key Laboratory of Natural Medicines, Jiangsu Key Laboratory of TCM Evaluation and Translational Research, Research Center for Traceability and Standardization of TCMs, Cellular and Molecular Biology Center, School of Traditional Chinese Pharmacy, China Pharmaceutical University, Nanjing 211198, P.R. China

* These authors contributed equally to this work.

✉Corresponding authors, Bo-Yang Yu, Email: boyangyu59@163.com; Jiangwei Tian, Email: jwtian@cpu.edu.cn

Supplementary tables

Table S1. The size, PDI and zeta potential of DBC NPs and Tf-DBC NPs (n=3).

Nanodrug	Size (nm)	PDI	Zeta potential (mV)
DBC NPs	118.4±3.4	0.224±0.02	-22.3±0.18
Tf-DBC NPs	127.2±5.8	0.218±0.02	-27.1±0.16

Table S2. Comparing the loading capacity and encapsulation efficiency of single and complete formation in Tf-DBC NPs (n=3).

Compounds	DHA		BSO		CellROX	
	Tf-D NPs	Tf-DBC NPs	Tf-B NPs	Tf-DBC NPs	Tf-C NPs	Tf-DBC NPs
Loading capacity	21.7%±2.1%	17.9%±2.3%	5.4%±1.3%	4.1%±0.9%	18.7%±3.6%	15.6%±1.1%
Encapsulation efficiency	88.9%±4.9%	76.4%±3.1%	45.5%±2.8%	33.3%±1.4%	80.3%±6.8%	71.4%±3.5%

Supplementary figures

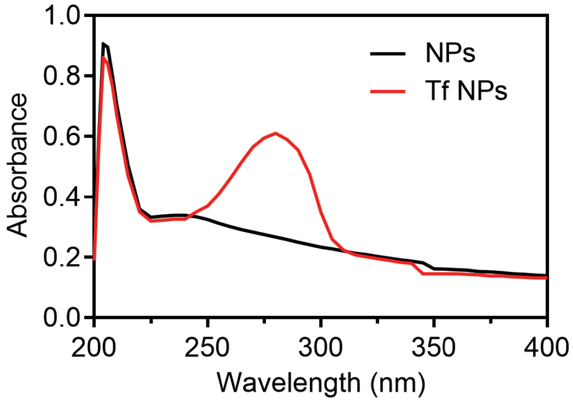

Figure S1. UV-VIS absorption spectra of NPs and Tf-NPs.

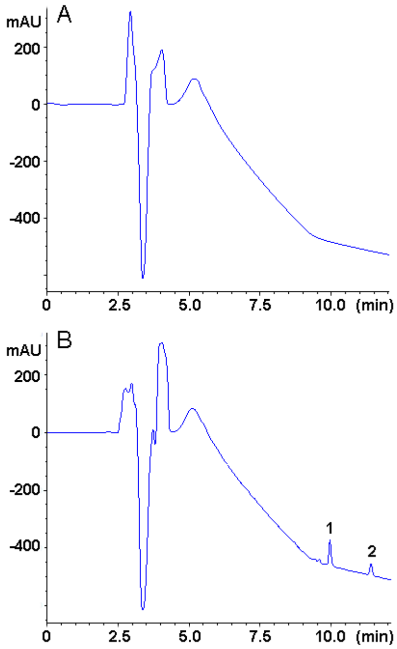

Figure S2. Representative chromatogram of (A) blank liposomal and (B) liposomal with DHA encapsulated. (wavelength: 215 nm, 1: DHA α-epimer, 2: DHA β-epimer).

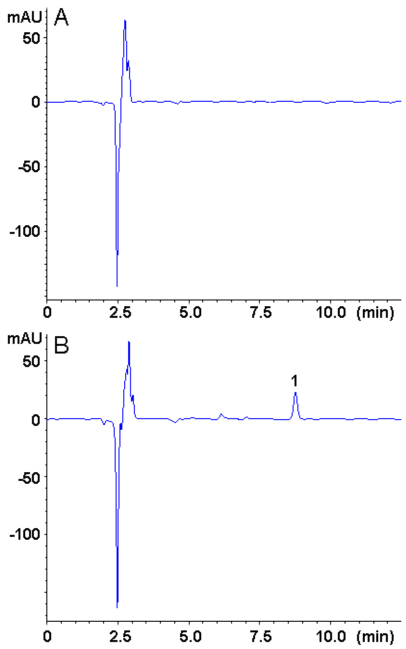

Figure S3. Representative chromatogram of (A) blank liposomal and (B) liposomal with BSO encapsulated. (wavelength: 335 nm, 1: BSO).

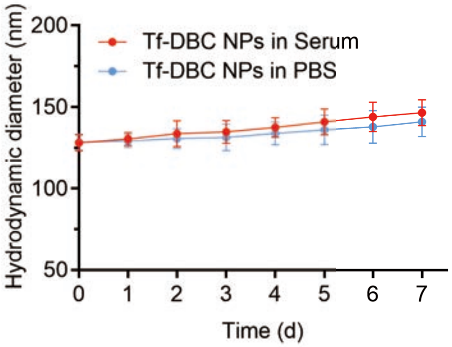

Figure S4. Size stability test of Tf-DBC NPs in DMEM with 10% FBS and PBS (PH=7.4).

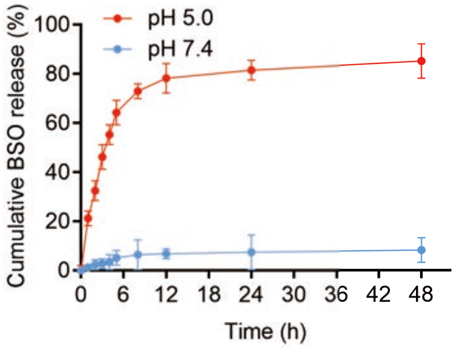

Figure S5. Accumulative release of BSO from Tf-DBC NPs at pH 7.4 and 5.0, respectively.

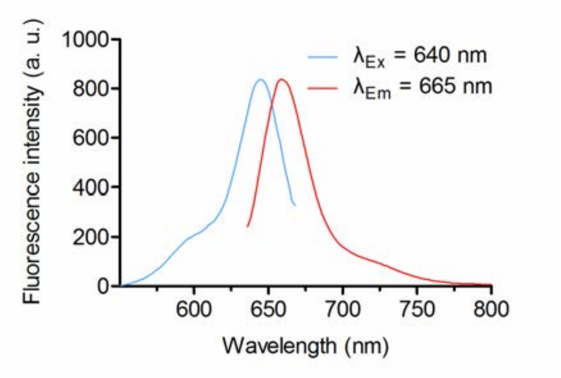

Figure S6. The fluorescent excitation and emission spectra of CellROX in the presence of H_2O_2.

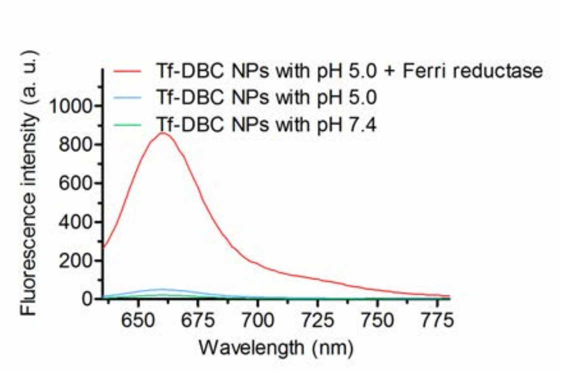

Figure S7. The fluorescence response of CellROX encapsulated in the complete Tf-DBC NPs in different environment.

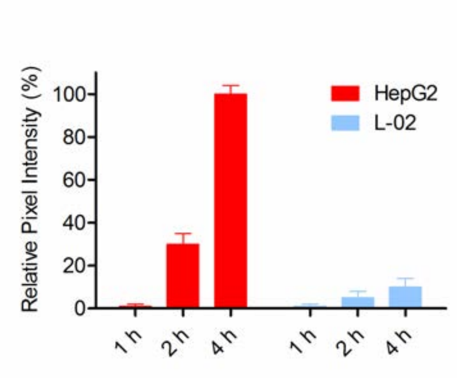

Figure S8. The pixel intensity of FITC calculated in HepG2 and L-02 cells with different incubation times. Data are means ± SD (n = 3).

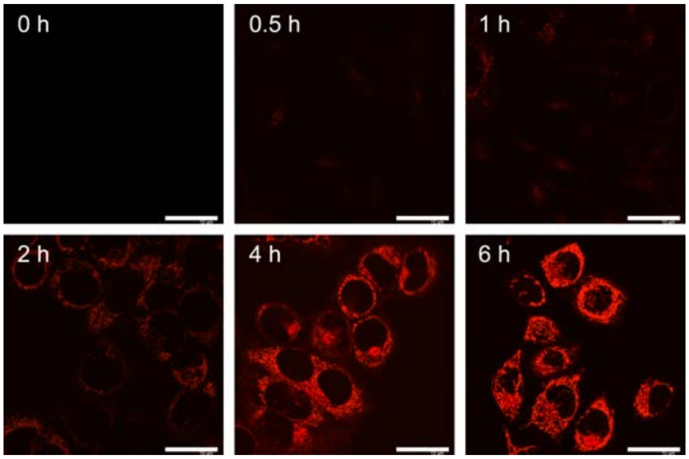

Figure S9. Confocal fluorescence images of HepG2 cells incubated with Tf-DBC NPs for different times. Scale bars: 10 μm.

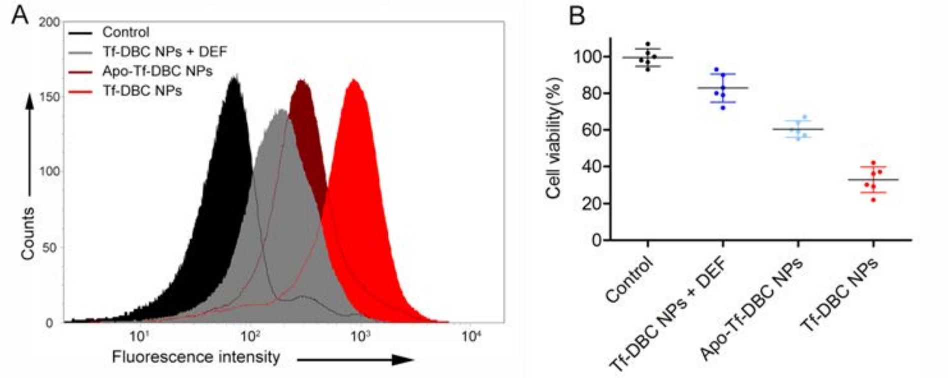

Figure S10. (A) Flow cytometric assay and (B) cell viability assay of HepG2 cells treated with Tf-DBC NPs, Apo-Tf-DBC NPs, and Tf-DBC NPs in the presence of deferiprone (DEF) as a scavenger of Fe(II).

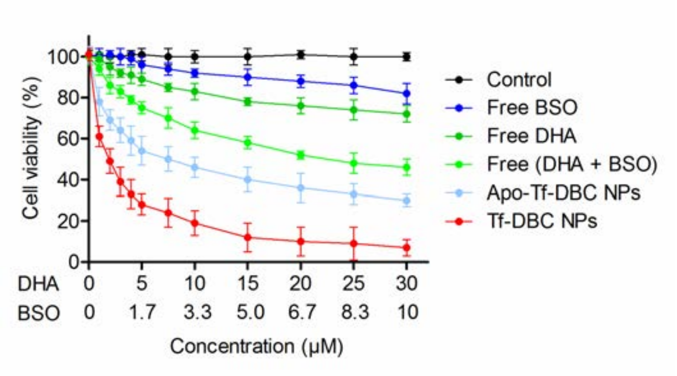

Figure S11. Cell viability assay of HepG2 cells treated with free DHA, free BSO, free DHA and BSO, Tf-DBC NPs and Apo Tf-DBC NPs.

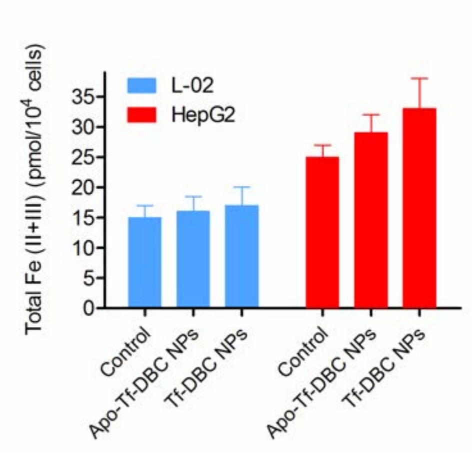

Figure S12. Comparison of content of iron ion measured by an iron colorimetric assay kit before/after L-02 and HepG2 cells were treated with different nanoparticles. Data are means ± SD (n = 3).

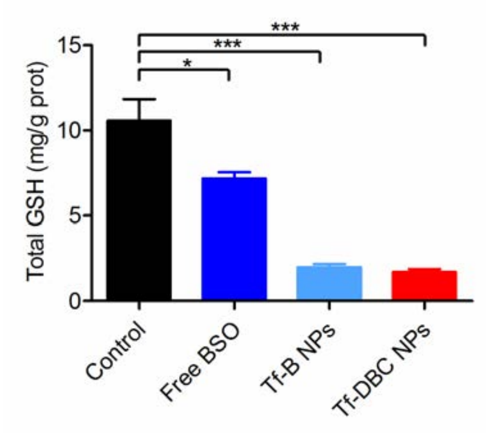

Figure S13. The GSH contents in HepG2 cells for different treated groups including control group, free BSO group, Tf-B NPs group, and Tf-DBC NPs group. Data are means ± SD (n = 3). **P*< 0.05, ***P*< 0.01, ****P*< 0.001.

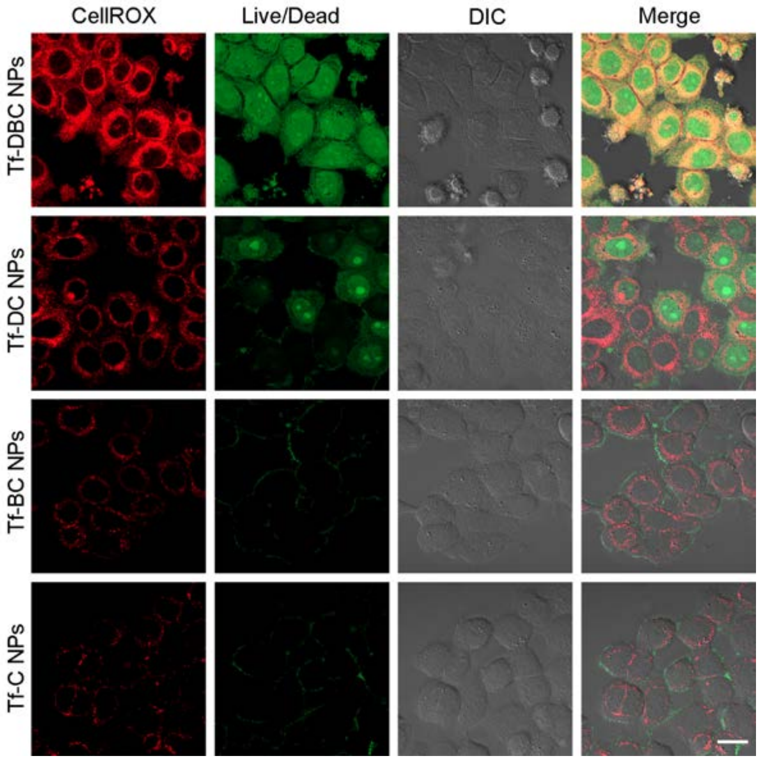

Figure S14. Confocal fluorescence images of HepG2 cells stained with LIVE/DEAD after incubated with Tf-C NPs, Tf-BC NPs, Tf-DC NPs and Tf-DBC NPs, respectively. Scale bars: 10 μm.

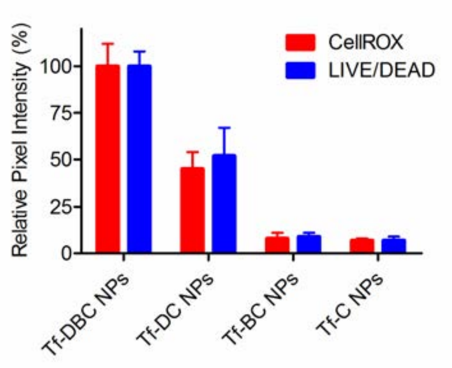

Figure S15. The pixel intensity of CellROX and LIVE/DEAD calculated in HepG2 cells after incubated with Tf-C NPs, Tf-BC NPs, Tf-DC NPs and Tf-DBC NPs, respectively. Data are means ± SD (n = 3).

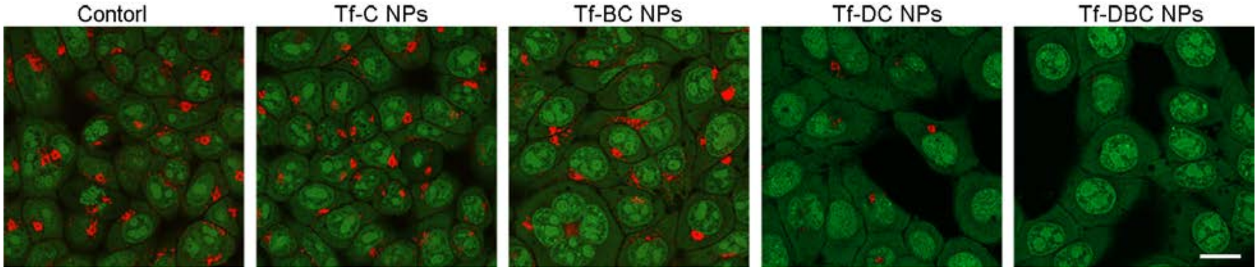

Figure S16. Lysosomal stability observed with confocal fluorescence images of AO-stained HepG2 cells after different treatments. Scale bars: 10 μm.

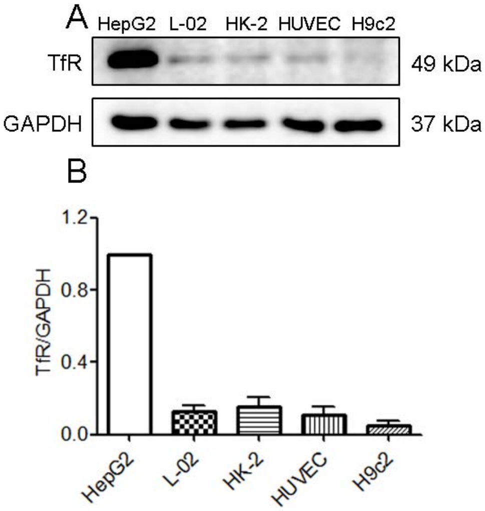

Figure S17. (A) The TfR expressions of different cells were tested by western blot using GAPDA as the loading control. (B) The semiquantitative analysis of TfR in different cells (n=3).

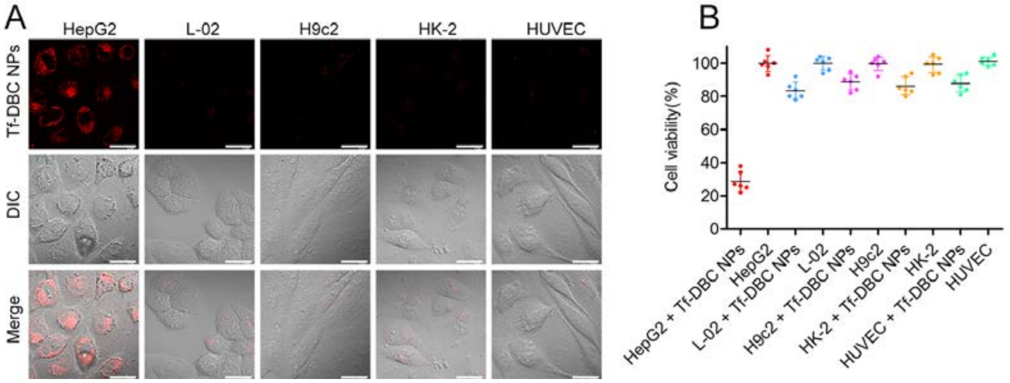

Figure S18. (A) Confocal fluorescence images of Tf-DBC NPs incubated HepG2, L-02, H9c2, HK-2, and HUVEC cells, respectively. (B) MTT assays for HepG2, L-02, H9c2, HK-2 and HUVEC cells incubated with Tf-DBC NPs(n=6).

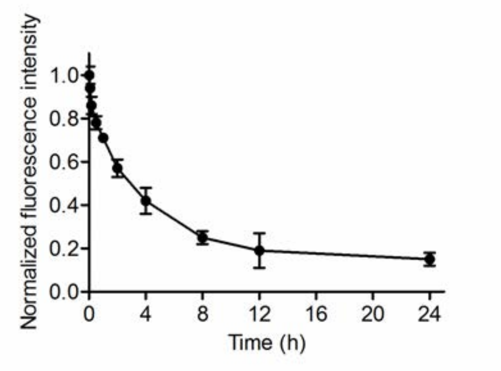

Figure S19. Blood circulation profile of Tf-Cy7 NPs (n=6).

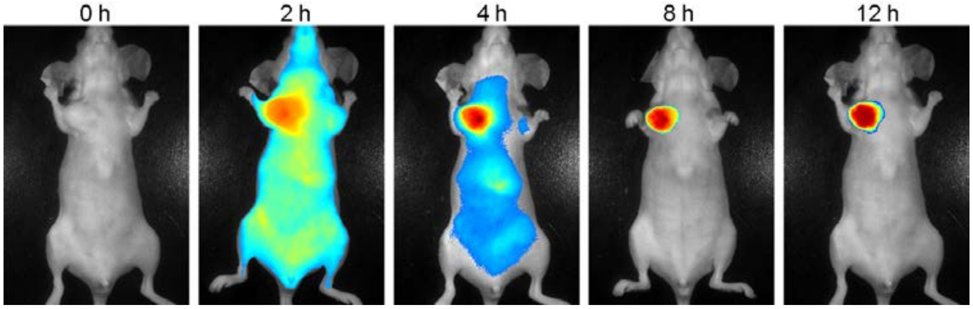

Figure S20. Time-dependent *in vivo* fluorescence images of subcutaneous HepG2 tumor-bearing mice after i.v. injection of Tf-Cy7 NPs.

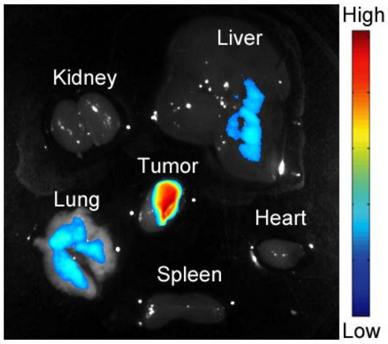

Figure S21. The *ex vivo* fluorescence images of tumor and other major organs collected after the mice were killed at 12 h post injection of Tf-Cy7 NPs.

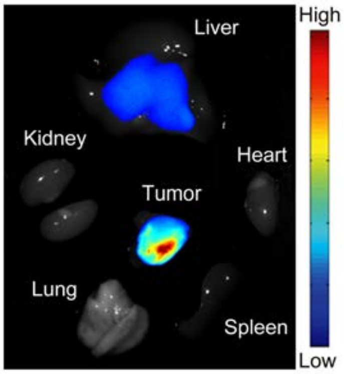

Figure S22. The *ex vivo* fluorescence images of tumor and other major organs collected after the mice were killed at 12 h post injection of Tf-DBC NPs.

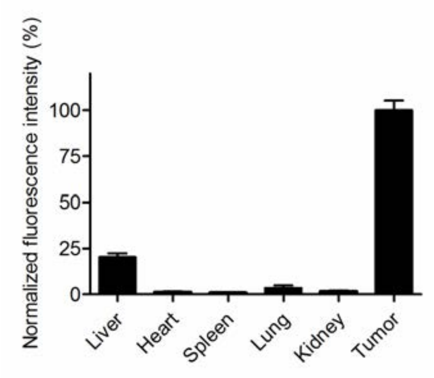

Figure S23. Semiquantitative biodistribution of Tf-DBC NPs in different organs and tumor in HepG2-tumor bearing mice (n=6).

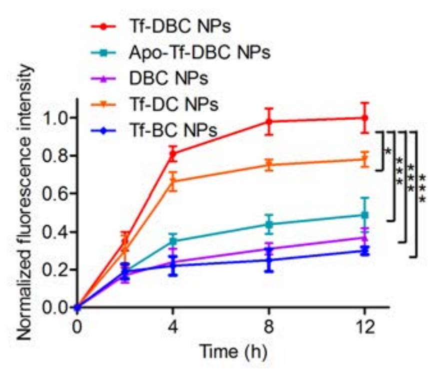

Figure S24. The normalized fluorescence intensity of intratumoral ROS after various treatments. Data are means ± SD (n = 6). **P*< 0.05*,* ***P*< 0.01*,* ****P*< 0.001.

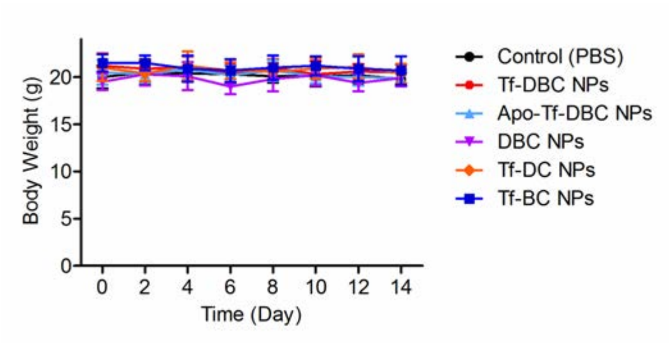

Figure S25. The body weight of mice after various treatments (n=6).

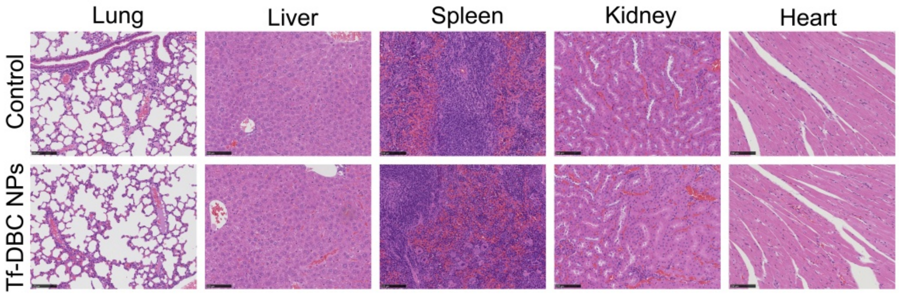

Figure S26. Representative H&E-stained histological sections of major organs after treatment with Tf-DBC NPs for *in vivo* toxicity assay.
